# Small molecule-driven LKB1 deacetylation is responsible for the inhibition of hepatic lipid response in NAFLD

**DOI:** 10.1016/j.jlr.2024.100740

**Published:** 2025-01-02

**Authors:** Weiwei Qin, Yu Ding, Wenhao Zhang, Lu Sun, Jianping Weng, Xueying Zheng, Sihui Luo

**Affiliations:** 1 Inflammation and Immune Mediated Diseases Laboratory of Anhui Province, School of Pharmacy, Anhui Medical University, Hefei, China; 2 Institute of Endocrine and Metabolic Diseases, The First Affiliated Hospital of USTC, Division of Life Sciences and Medicine, University of Science and Technology of China, Hefei, China; 3 Department of Endocrinology, The First Affiliated Hospital of Anhui Medical University, Hefei, China

**Keywords:** AMPK, Hepatic lipid response, HFHC, MCD, NASH, Tranilast

## Abstract

Nonalcoholic fatty liver disease (NAFLD) is a progressive condition characterized by ectopic fat accumulation in the liver, for which no FAD-approved drugs currently exist. Emerging evidence highlights the role of liver kinase B1 (LKB1), a key metabolic regulator, has been proposed in NAFLD, particularly in response to excessive nutrient levels. However, few agents have been identified that can prevent the progression of nonalcoholic steatohepatitis (NASH) by targeting LKB1 deacetylation. Through comprehensive screening of our in-house chemical library, we identified tranilast, a small molecule with remarkable inhibitory efficacy against lipid deposition induced by palmitic acid/oleic acid (PO). In this study, we investigated the novel biological function and mechanism of tranilast in regulating hepatic lipid response in NAFLD, focusing on its role in LKB1 deacetylation within hepatocytes. Our findings demonstrate that tranilast effectively reduced hepatic steatosis, inflammation, and fibrosis in NASH models induced by high-fat and high-cholesterol (HFHC) and methionine choline-deficient (MCD) diets. Mechanistic analysis using RNA sequencing revealed that tranilast mitigated hepatic lipid response by promoting LKB1 deacetylation and activating AMPK. Notably, in vivo experiments showed that the beneficial effects of tranilast in MCD diet-induced NASH model were reversed by the compound C (C-C), a known AMPK inhibitor, confirming that tranilast’s effects on hepatic lipid response are mediated through the AMPK pathway. In summary, tranilast inhibits hepatic lipid response in NAFLD through LKB1 deacetylation, providing robust experimental evidence for the role of LKB1 in NAFLD. These findings position tranilast as a promising therapeutic candidate for the pharmacological management of metabolic diseases.

## Graphical Abstract

**Figure:**
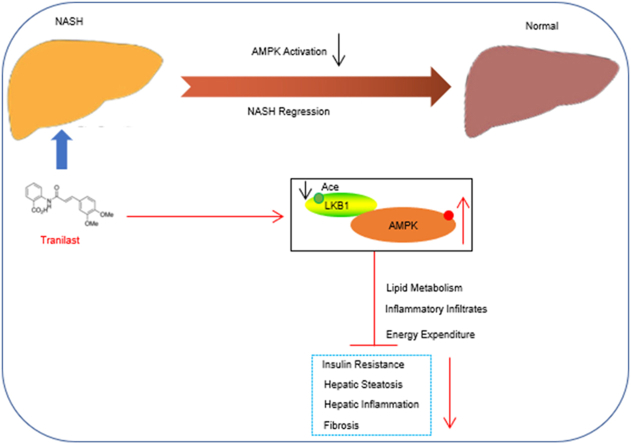

Nonalcoholic fatty liver disease (NAFLD) results from free fatty acid (FFA) metabolic dysfunction in the liver and encompasses a spectrum of hepatic conditions, ranging from nonalcoholic fatty liver (NAFL) to the more severe and malignant form, nonalcoholic steatohepatitis (NASH). NASH represents a progressive manifestation of metabolic syndrome and serves as a critical risk factor for fibrosis, cirrhosis, and hepatocellular carcinoma (HCC). With lifestyle changes and increased prevalence of obesity, NAFLD has become a significant public health epidemic and the leading chronic liver disease, closely linked to insulin resistance, type 2 diabetes mellitus (T2DM), and obesity (1). Approximately 20% of patients with NAFLD progress to NASH, which is marked by overnutrition-induced liver injury, chronic hepatic inflammation, and progressive fibrosis alongside steatosis (2, 3). Interestingly, NASH also affects lean individuals, driven by factors such as specific nutrient overconsumption, visceral fat deposition, genetic predisposition, dyslipidemia, or hepatic lipodystrophy (4). Unlike simple hepatic steatosis, NASH frequently evolves into severe liver conditions, including cirrhosis and HCC, and is a leading cause of liver transplantation and liver-related mortality worldwide (5). Despite considerable research efforts, the pathogenesis of NAFLD remains poorly understood, and no Food and Drug Administration (FAD)-approved medications or licensed therapies are currently available for clinical use. This underscores an urgent need to identify or develop novel therapeutic agents and effective treatment strategies for this highly prevalent metabolic disorder.

The high-fat and high-cholesterol (HFHC) diet model closely resembles human NASH, representing a state of overnutrition-induced hepatic steatosis, advanced inflammation, and fibrosis. It is widely recognized as a reliable NASH model (6, 7). In clinical practice, several drugs are commonly used to manage NASH, including hypoglycemic agents, lipid-lowering drugs, and insulin sensitizers (8). However, the efficacy, quality, and safety of these treatments remain uncertain, and their precise role in preventing and treating NAFLD is not well established. These drugs typically focus on managing hyperglycemia or hyperlipidemia, rather than directly targeting NAFLD. In response to this gap, a more simplified model of fatty liver disease, the methionine-choline deficient (MCD) diet model, was developed. This model has proven valuable for studying drug candidates specifically aimed at NASH. Additionally, the MCD diet model is considered a gold standard for non-nutritional NASH and is a classical inflammatory model used in several clinical trials (9, 10). Although animal models of NASH are now available, the absence of effective therapeutic drugs has hindered a comprehensive understanding of the molecular mechanisms underlying diet-induced NASH.

The progression of NAFLD is closely linked to the excessive accumulation of fatty acids and lipotoxic metabolites (11, 12). Hepatic steatosis, the earliest stage of NAFLD, arises when triglyceride (TG) synthesis exceeds TG disposal in the liver (13, 14). The excessive lipid accumulation in hepatocytes increases lipotoxic FFAs and promotes the release of proinflammatory cytokines through oxidative stress (15). Moreover, NAFLD often coexists with hepatic insulin resistance, which heightens the risk of fasting hyperglycemia and T2DM (16). Recent studies, including our own, have shown that NLRP3 is a direct factor driving hepatic insulin resistance and NAFLD (17). Although the roles of hepatic TG accumulation and insulin resistance are well established in NAFLD, the molecular mechanisms underlying hepatic lipid response remain unclear.

Tranilast, a derivative of a tryptophan metabolite, was developed and approved as an anti-allergic drug in the 1980s (18). Clinically, tranilast is a small-molecule drug known for its high safety profile, good bioavailability, and favorable drug-like properties. It is used to treat various inflammatory conditions, including bronchial asthma, atopic dermatitis, allergic conjunctivitis, and hypertrophic scars (19). Additionally, tranilast has demonstrated therapeutic effects in type-I allergic diseases and inflammatory conditions, such as allergic rhinitis, acute colitis, and atherosclerosis (20, 21, 22, 23). Several studies have suggested that tranilast may offer protective benefits against pulmonary injury and fibrosis (24, 25), indicating its broad therapeutic potential. Moreover, tranilast has been shown to reduce hepatic fibrosis, inflammation, and Kupffer cell recruitment in a rat model of NASH (26). However, this research primarily focused on liver-resident macrophages (Kupffer cells) rather than hepatocytes. Although tranilast’s protective effects have been studied in various diseases, its precise therapeutic function and underlying mechanisms in regulating hepatic lipid response in hepatocytes remain unclear.

The present study aims to investigate, for the first time, the effects and molecular mechanisms of tranilast in a mouse model of NASH induced by HFHC and MCD diets, with a particular focus on hepatocytes. These two mouse models closely mimic the metabolic features of human NASH, including lipid accumulation, hepatic inflammation, and fibrosis. Mechanistically, RNA sequencing (RNA-seq) analysis revealed that tranilast combats hepatic lipid accumulation in NAFLD by deacetylating liver kinase B1 (LKB1) and promoting AMP-activated protein kinase (AMPK) activation. We expect that this study will provide robust experimental evidence regarding the role of LKB1 deacetylation in the treatment of NASH and suggest that tranilast may be a promising new drug for the pharmacological management of metabolic syndrome.

## Materials and Methods

### Reagents

Tranilast (purity ≥ 98%, #T2690), Compound C (C-C, #T6146), and Pim1 (#T5093) were all purchased from Topscience Co., Ltd. STO-609 (#CSN19242) was purchased from CSNpharm Co., Ltd.. The bicinchoninic acid (BCA) protein assay kit (#23225) and BODIPY (493/503, #D3922) were sourced from Thermo Fisher Scientific. Palmitic acid (PA, #27734), oleic acid (OA, #O1008), oil red O (ORO, #O1391), fatty acid-free bovine serum albumin (BSA, #SRE0098), and collagenase type IV (#C5138) were purchased from Sigma-Aldrich. TRIzol reagent (#AM9738) and 4′,6-diamidino-2-phenylindole (DAPI, #10236276001) were purchased from Invitrogen. Insulin (#P3375) and PMSF (#ST506) were purchased from Beyotime Biotechnology. All powder reagents were dissolved in dimethyl sulfoxide (DMSO, working concentration <0.1%) to prepare stock solutions.

### Cell culture and primary hepatocyte and Kupffer cells isolation

L02 cells, a human hepatocyte cell line, were obtained from the Type Culture Collection of the Chinese Academy of Sciences (Shanghai, China). The cells were cultured in DMEM (Gibco, #11965-092) supplemented with 10% (*v/v*) fetal bovine serum (FBS, Gibco, #12483-020) and 1% penicillin-streptomycin (PS, Gibco, #15140-122) in a humidified incubator with 95% air and 5% CO2 at 37°C. Cells were treated under the specified conditions according to the experimental requirements.

We isolated primary hepatocytes from the livers of male C57BL/6J mice (8–10 weeks old, 24–28 g), following a previously described protocol (17, 27). Briefly, mice were anesthetized via intraperitoneal injection of pentobarbital sodium, and their abdominal cavities were opened using surgical scissors. The livers were perfused in situ with a pronase/collagenase solution. Once perfused, the livers were carefully removed, minced, and dissociated by gentle shaking. The resulting cell suspension was filtered through sterile gauze and centrifuged at 200 *g* for 2 min at 4°C. This process yielded a pellet (hepatocytes) and a supernatant (Kupffer cells). Hepatocytes were seeded onto 60-mm dishes at a density of 1 × 10^6^ cells per dish in William's E medium supplemented with 10% FBS and 1% PS. After 4 h, the medium was supplemented with dexamethasone (100 nM) and insulin (1 nM). Hepatocyte identity was confirmed using periodic acid-Schiff (PAS) staining, and cell viability was assessed via trypan blue exclusion. Kupffer cells were isolated using anti-F4/80 MicroBeads UltraPure (#130-110-443, Miltenyi Biotec) and LS columns (#130-042-401, Miltenyi Biotec), following the manufacturer’s instructions. After a 24-h incubation, primary hepatocytes and Kupffer cells were stimulated under conditions specified in the figure legends. L02 cells and primary hepatocytes were treated with fatty acid-free bovine serum albumin (BSA, 0.5%) or a mixture of palmitic acid (PA, 0.5 mM) and oleic acid (OA, 1.0 mM) (PO) in the presence or absence of tranilast at the indicated concentrations for 12 h before sample collection.

### AMPK and LKB1 mutation and knockdown

To inactivate AMPK, we introduced a mutation substituting threonine 172 with alanine (T172A), as previously described (28). For LKB1 acetylation, we introduced mutations at D194, replacing each with alanine (D194A), as detailed in a previous study (29). We purchased AMPKα1/2-specific siRNA duplex (sc-45312) and control siRNA (sc-37007) from Santa Cruz Biotechnology and transfected the cells using transfection reagent (sc-29528) according to the manufacturer’s instructions. LKB1 siRNA (STK11 siRNA: GCCAACGTGAAGAAGGAAATT) was obtained from GenePharma.

### Animals and experimental protocol

Male C57BL/6J mice (6–8 weeks old, 18–22 g) were obtained from the Model Animal Research Center of Nanjing University (license number: SCXK (Su) 2023-0009; Nanjing, China). Prior to the experiments, the animals underwent a 1-week acclimatization period in a specific pathogen-free facility, where they were randomly assigned to feeding conditions. The mice were housed in plastic cages at a temperature of 21 ± 2°C, with a 12-h light/dark cycle and 50 ± 10% relative humidity. All animal procedures were approved by the Animal Ethical and Welfare Committee of the Center for Animal Experiments at the University of Science and Technology of China (USTC) and the Animal Care and Use Committee of the First Affiliated Hospital of USTC (USTCACUC212401038; February 2, 2021). The study adhered to all relevant ethical guidelines for animal research, as outlined in the National Institutes of Health Guide for the Care and Use of Laboratory Animals (NIH Publications No. 8023, revised 1978). We made every effort to reduce the number of animals used and minimize their suffering.

For the HFHC diet-induced NASH model, mice were randomly assigned to different groups, ensuring similar plasma glucose levels and body weights across groups. They were fed an HFHC diet (D09100310, Research Diets, NJ, USA) for 16 weeks. Mice in the control group were given a standard normal chow diet (D10001, Research Diets). During the final 8 weeks of the study, mice received either sodium carboxymethylcellulose (Na-CMC) or tranilast (25 or 50 mg/kg) once daily, with or without the HFHC diet. The selected tranilast doses were based on prior research (23) and converted clinical dosages for adults. Tranilast was suspended in Na-CMC (0.5%) solution using ultrasonication and administered orally at a volume of 10 ml/kg. At the study's conclusion, liver and adipose tissues from the mice were harvested for further analysis.

For the MCD diet-induced NASH model, mice were grouped and fed an MCD diet (A02082002BR, Research Diets) ad libitum for 4 weeks. Control mice were provided a methionine- and choline-supplemented diet (MCS; A02082003B, Research Diets). Tranilast was administered intragastrically (*i.g.*) daily at the specified doses for the entire duration of the experiment, while Compound C (C-C), an AMPK inhibitor, was injected intraperitoneally (*i.p.*) every 2 days at the indicated doses over the 4-week period. All groups received an equal volume of Na-CMC as vehicle control. After the final treatment, liver tissues were collected from the mice for subsequent experiments.

### Metabolic studies

Prior to initiating the experiments, mice in the designated groups were fasted for 14–16 h for oral glucose tolerance tests (OGTTs) or for 4–6 h during the daytime for insulin tolerance tests (ITTs). In the OGTTs, after fasting, mice were orally administered D-glucose (2.0 g/kg, analytical reagent). For the ITTs, mice were acclimatized for 1 week prior to the experiment. After fasting, the mice were intraperitoneally injected with insulin (1.0 U/kg). Tail blood glucose levels were measured at 0, 15, 30, 60, 90, and 120 min using the OneTouch Ultra blood glucose monitoring system. The area under the curve (AUC) was calculated and presented in the figures. For metabolic cage studies, after 16 weeks of HFHC diet feeding, mice in the indicated groups were acclimatized to the cages (Columbus Instruments) for 1 day. Over the following 72 h, VO_2_, VCO_2_, heat production, and food intake were measured using a comprehensive laboratory animal monitoring system (CLAMS).

### Lipid accumulation assay

For BODIPY staining and fluorescence signal quantification, L02 cells were seeded in confocal dishes overnight and incubated with PO in the presence or absence of tranilast at the specified concentrations for 12 h. The cells were then fixed with paraformaldehyde and stained with BODIPY (0.5 μM) in PBS for 30 min at room temperature. Images were captured using the ImageXpress Micro Confocal (LSM080, Zeiss). Image acquisition settings were standardized throughout the process. Fluorescence quantification was performed by determining the relative fluorescent units of lipid droplets using ImageJ software.

### Liver histological analysis

Liver tissues were dissected and fixed in 4% (*w/v*) paraformaldehyde. After embedding in paraffin, the tissues were sectioned into 5 μm thick slices. For histological evaluation, sections were stained with H&E and Oil Red O following standard protocols according to the manufacturer’s instructions. Images were captured and analyzed using an optical microscope.

### Quantitative real-time PCR (qPCR)

L02 cells and primary hepatocytes were incubated with a mixture of PO, with or without tranilast at the specified concentrations, for 12 h. Total RNA was isolated from the cells or liver tissues of mice using TRIzol, following the manufacturer’s protocol. RNA concentration was determined using a Nanodrop at 260 and 280 nm. Subsequently, 1.0 μg of RNA was reverse transcribed into cDNA using a Reverse Transcriptase Kit (#RR037A, Takara) according to the standard protocol. qPCR was performed using a SYBR Green Premix Kit (#RR820A, Takara) on a LightCycler 96 System (Roche). The reaction conditions were as follows: 95°C for 3 min, followed by 35 cycles of 95°C for 10 s, and 60°C for 30 s β-action mRNA was used as an internal reference gene. The relative mRNA expression levels of target genes were calculated using the 2^−ΔΔCt^ method. The primer sequences used in the study are listed in Supplemental Table S1 (Sangon Biotech).

### Immunoblot analysis

For total protein analysis, cells were lysed in ice-cold RIPA buffer supplemented with 1.0 mM PMSF. For liver tissue homogenates from mice, the liver was dissected into small sections (40 mg) and homogenized in ice-cold RIPA buffer, also containing 1.0 mM PMSF. After incubating on ice for 40 min, we obtained the proteins by centrifugation at 12,000 *g* for 20 min at 4°C. Protein concentrations were determined using the BCA protein assay kit. Equal amounts of protein (approximately 30.0 μg) were loaded onto SDS-PAGE gels, and the proteins were transferred to a PVDF membrane (IPVH00010, Millipore) via electroblotting.

We used the following primary antibodies: anti-phospho-IKKβ (#2697, 1:1000), anti-NF-κB-p65 (#8242, 1:1000), anti-phospho-NF-κB-p65 (#3033, 1:1000), anti-Akt (#4691, 1:1000), anti-phospho-Akt (Ser473, #2859, 1:1000), anti-phospho-GSK3β (#5558, 1:1000), anti-AMPKα (#5831, 1:1000), anti-phospho-AMPKα (#2535, 1:1000), anti-Col1a1 (#72026, 1:1000), and anti-α-SMA (#19245, 1:1000) from Cell Signaling Technology; anti-IKKβ (#ab124957, 1:1000) from Abcam; anti-GSK3β (#22104-1-AP, 1:1000), anti-FASN (#10624-2-AP, 1:1000), anti-ACLY (#67166-1-Ig, 1:1000), anti-PPARγ (#16643-1-AP, 1:500), anti-LKB1 (#10746-1-AP, 1:500), anti-GAPDH (#60004-1-Ig, 1:2000), and anti-HRP-coupled secondary antibody (#SA00001-1 or #SA00001-2, 1:5000) from Proteintech; anti-Ac-Lys (#sc-32268, 1:100) from Santa Cruz Biotechnology; and anti-SREBF1 (#557036, 1:1000) from BD Biosciences.

After overnight incubation with the primary antibodies at 4°C, the membranes were incubated with the HRP-coupled secondary antibody. We detected the protein bands using the Tanon chemiluminescent substrate system (Tanon).

### Immunoprecipitation (IP)

L02 cells (2 × 10^5^ cells/ml) were seeded into 10-mm dishes, with three dishes per group for biological replicates, and incubated for 24 h. At 80% confluency, we treated the cells with tranilast (30 μM) in the presence or absence of PO for 12 h. After treatment, we lysed the cells in IP lysis buffer on ice for 30 min and obtained the proteins by centrifugation at 12,000 *g* for 15 min at 4°C. We incubated the proteins with 1.0 μg of the indicated antibody at 4°C overnight, followed by precipitation with protein A/G-agarose (#88802, Thermo Fisher Scientific) according to the manufacturer’s instructions. We subjected the immunoprecipitated proteins to SDS-PAGE and performed immunoblotting using the indicated antibodies. The next steps followed the standard immunoblot analysis procedure.

### Immunofluorescence staining

We fixed and embedded liver tissue sections from mice according to standard procedures and the manufacturer’s instructions. The samples were permeabilized with 0.1% Triton X-100 for 10 min and blocked with BSA buffer at room temperature for 30 min. We then incubated the samples overnight at 4°C with the anti-F4/80 antibody (#ab6640, 1:200, Cambridge). Afterward, the samples were incubated with the appropriate fluorescence-conjugated secondary antibody. Finally, we stained the nuclei with DAPI for 10 min. Images were captured using fluorescence microscopy.

### Biochemical assay and Enzyme-linked immunosorbent assay (ELISA) analysis

We evaluated the supernatants from the serum and tissue samples of mice in each group for alanine aminotransferase (ALT, #E-BC-K235-M, Elabscience Biotech), aspartate aminotransferase (AST, #E-BC-K236-M), total cholesterol (TC, #E-BC-K109-M), triglycerides (TG, #E-BC-K261-M), and low-density lipoprotein cholesterol (LDL-C, #E-BC-K205-M) according to the manufacturer's standardized instructions. For the measurement of intracellular TG and TC, L02 cells or primary hepatocytes were cultured in DMEM supplemented with 10% FBS. Once the cells reached 80% confluence, we treated them with tranilast, STO-609, or Pim1, with or without PO, for 12 h as described previously. We then collected the cell supernatants for TG and TC analysis using the indicated kits, normalizing the results to the total cellular protein content. To assess insulin levels, we collected serum from mice in each group treated with the mentioned agents. We measured insulin levels (#E-EL-M1382c) using ELISA, following the manufacturer's standardized instructions.

### RNA seq and Kyoto encyclopedia of genes and genomes (KEGG) pathway enrichment analysis

Total RNA was isolated from the liver tissues of the indicated mice using TRIzol reagent, and the process was carried out by the Columbia Genome Center. Paired-end libraries were synthesized using the TruSeq RNA Sample Preparation Kit, following the TruSeq RNA Sample Preparation Guide. Poly-A pull-down was applied to enrich the mRNA from the total liver RNA. Sequencing of the library was performed on the Illumina HiSeq X-ten platform. The detailed procedures for data analysis were provided by GENE DENOVO. For KEGG pathway enrichment analysis, differentially expressed genes (DEGs) were analyzed using Fisher’s exact test. DEGs were identified using the R package limma, with a |log2(Fold Change)| threshold of 1.5. Functional enrichment analysis was performed using the ClusterProfiler R package. Pathways with a *P*-value ≤0.05 were considered significantly enriched.

### Statistical analysis

Data analysis was conducted using GraphPad Prism version 9.0 (GraphPad Software). All data are presented as the mean ± standard error of the mean (SEM). Differences between two groups with parametric data were assessed using a Student's *t* test. A one-way ANOVA was used for parametric data involving multiple comparisons. Post hoc tests, including Bonferroni’s or Tamhane’s T2 (M), were performed based on the homogeneity of variance. For data with skewed distributions, the Mann-Whitney U test and Kruskal-Wallis test were used for two-group and multiple-group comparisons, respectively. *P* < 0.05 was considered statistically significant.

## Results

### Identification of tranilast as a regulator of lipid accumulation and inflammation

**Fig. 1:**
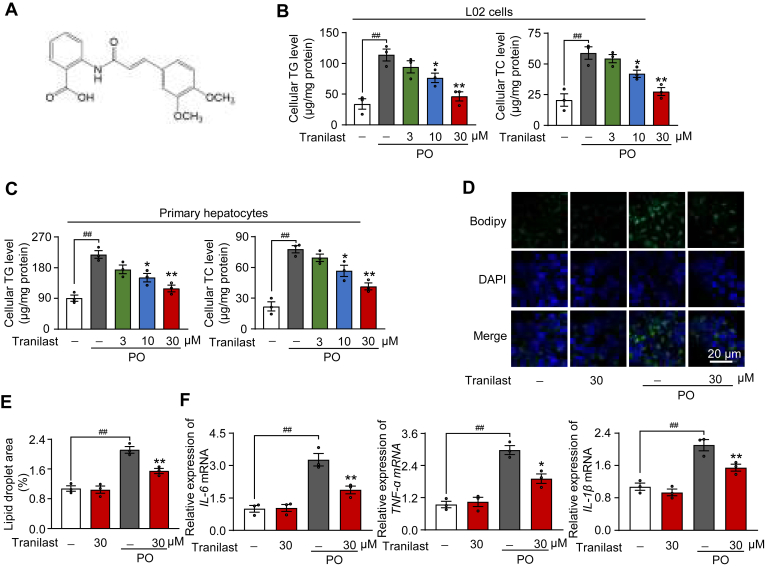
Identification of tranilast as a regulator of lipid accumulation and inflammation. A: Chemical structure of tranilast. B, C: TG and TC contents of L02 cells (B) and primary hepatocytes (C) in the indicated groups. D, E: Representative images of BODIPY staining (D) and quantification of fluorescence (E) in PO-induced L02 cells. (F) qPCR analysis of mRNA levels for *IL-6*, *TNF-α* and *IL-1β* in PO-induced L02 cells. Data are presented as mean ± SEM. n = 3 biologically independent experiments. ^##^*P* < 0.01, ∗*P* < 0.05, ∗∗*P* < 0.01 *vs*. The indicated group or the PO treatment group. One-way ANOVA was used for statistical analysis (B, C, E, and F). PO, palmitic acid and oleic acid; TG, triglyceride; TC, total cholesterol.

To address a range of metabolic diseases, we developed small lead compounds and created a drug library in our laboratory. Through screening this optimized in-house library, we identified tranilast, an anti-allergic clinical drug, as a potential repurposed drug candidate using a lipid accumulation assay in L02 cells. This strategy revealed a diverse set of small compounds capable of reducing lipid accumulation in L02 cells exposed to a PO challenge. Among these, tranilast (Fig. 1A) emerged as a particularly potent drug, demonstrating an optimal safety, tolerability, and pharmacokinetic profile (19, 23, 26). Given its promising effects, we further explored tranilast's impact on lipid levels and inflammation in vitro. Notably, tranilast inhibited PO-induced cellular lipid accumulation in a concentration-dependent manner, as shown by the reduced TG and TC levels in L02 cells and primary hepatocytes (Fig. 1B, C). Cell viability assays confirmed that tranilast did not induce cytotoxicity at concentrations below 30 μM in this experimental system (Supplemental Fig. S1). Additionally, BODIPY staining confirmed that tranilast reduced PO-induced lipid accumulation in L02 cells (Fig. 1D, E). We also examined the mRNA expression of several genes involved in inflammatory responses. As shown in Fig. 1F, tranilast significantly decreased the mRNA levels of *IL-6*, *TNF-α*, and *IL-1β* in PO-induced L02 cells. Taken together, these results demonstrate that tranilast effectively suppresses cellular lipid accumulation and inflammation.

### Tranilast protects against obesity and insulin resistance in mice induced by HFHC diet

**Fig. 2:**
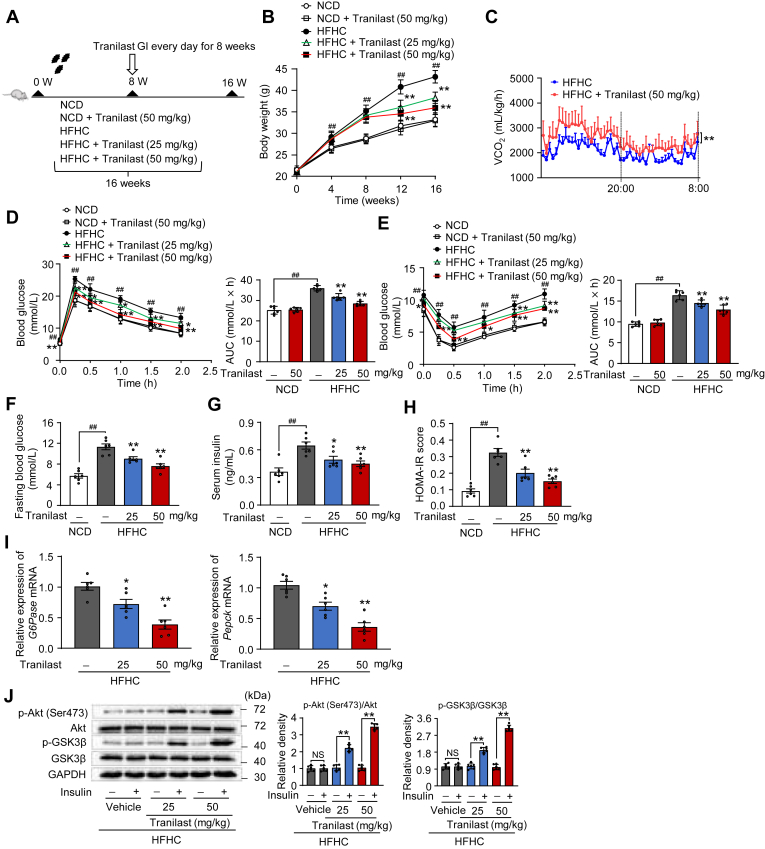
Tranilast protects against obesity and insulin resistance in mice induced by HFHC diet. 8-week-old male C57BL/6J mice were randomly grouped (n = 6 per group). Mice were given ad libitum access to water and different types of diets. Vehicle or tranilast (25 or 50 mg/kg) was administrated to mice by gastric irrigation (GI) once daily for 8 weeks. Mice were finally sacrificed and subjected to various analyses as indicated below. A: Schematic of the experimental procedure. B: Body weight monitored every week. C: Energy expenditure recorded using CLAMS and the oxygen consumption rate (VO_2_). D, E: OGTT (D) and ITT (E) performed and quantified as AUC. F-H: Levels of fasting blood glucose (F), plasma insulin (G), and HOMA-IR score (H). I: qPCR analysis of mRNA expression of *G6Pase* and *Pepck* from the liver sections. J: Immunoblot analysis of the indicated proteins in the insulin signaling pathways of the liver tissues. Data are presented as mean ± SEM (n = 6). ^##^*P* < 0.01, ∗*P* < 0.05, ∗∗*P* < 0.01 *vs*. the indicated groups or the HFHC treatment group. One-way ANOVA was used for statistical analysis in (B, D-I). Student's *t* test and Mann-Whitney U test was used for statistical analysis in (C, J). AUC, area under the curve; HFHC, high-fat high-cholesterol; ITT, insulin tolerance test; NCD, normal chow diet; OGTT, oral glucose tolerance test.

Building on the beneficial effects of tranilast, we next examined its impact on HFHC diet-induced hepatic lipid response in vivo. After 8 weeks of HFHC diet feeding, mice were treated with either vehicle or tranilast (25 and 50 mg/kg) via daily oral gavage for an additional 8 weeks (Fig. 2A). Throughout the treatment period, tranilast showed no evident toxicity, and the treatment groups exhibited comparable food intake and absorption (Supplemental Fig. S2A, B). Notably, tranilast significantly reduced body weight in HFHC diet-fed mice (Fig. 2B). We also assessed the effects of tranilast on adipose tissue weights, including epididymal white adipose tissue (eWAT), inguinal white adipose tissue (iWAT), and brown adipose tissue (BAT), in the context of the HFHC diet-induced hepatic lipid response. Remarkably, tranilast reduced adipose tissue content in this model (Supplemental Fig. S2C, D). To explore the mechanisms underlying tranilast's effects on obesity, we evaluated energy expenditure. As anticipated, tranilast significantly increased the respiratory quotient in HFHC diet-fed mice (Fig. 2C), sindicating enhanced energy expenditure.

Additionally, tranilast improved glucose clearance and insulin sensitivity in HFHC diet-fed mice, as evidenced by enhanced glucose tolerance and reduced insulin resistance (Fig. 2D, E). Interestingly, tranilast had minimal effects on body weight, glucose tolerance, and insulin sensitivity in normal chow diet (NCD)-fed mice (Fig. 2B, D, E). Consistently, tranilast treatment lowered fasting blood glucose levels, serum insulin levels, and HOMA-IR values compared to the HFHC diet-challenged mice (Fig. 2F–H). Furthermore, tranilast significantly reduced the mRNA expression of gluconeogenesis-related genes *G6Pase* and *Pepck* (Fig. 2I). Moreover, tranilast enhanced the insulin signaling pathway in HFHC diet-induced mice, as indicated by the increased phosphorylation of Akt and GSK3β (Fig. 2J). Collectively, these findings demonstrate that tranilast alleviates obesity and insulin resistance in mice subjected to an HFHC diet.

### Tranilast inhibits HFHC diet-induced hepatic steatosis in mice

**Fig. 3:**
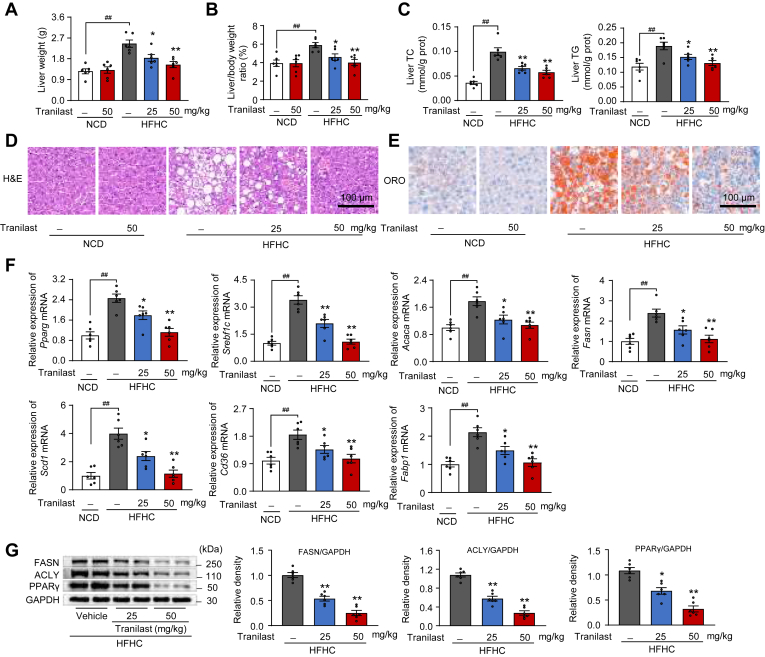
Tranilast inhibits HFHC diet-induced hepatic steatosis in mice. Same mice as in Fig. 2 were used. A, B: Liver weight (A) and ratio of liver weight to body weight (B). C: Levels of TC and TG in the liver tissues. D, E: Representative images of H&E (D) and ORO (E) staining in the liver sections. Scale bar, 100 μm. F: qPCR analysis of mRNA expression of fatty acid metabolism-related genes from liver tissues. G: Immunoblot analysis of proteins involved in lipid metabolism in liver tissues. Data are presented as mean ± SEM (n = 6). ^##^*P* < 0.01, ∗*P* < 0.05, ∗∗*P* < 0.01 *vs*. the indicated groups or the HFHC treatment group. One-way ANOVA was used for statistical analysis (A–C, F, and G). HFHC, high-fat high-cholesterol; ORO, oil red O; TG, triglyceride; TC, total cholesterol.

Hepatic steatosis is a common and severe complication associated with obesity and insulin resistance, often persisting throughout the progression of these conditions (7, 17). Therefore, we investigated the effects of tranilast on hepatic steatosis and lipid metabolism in this model. Hence, we determined the effects of tranilast on hepatic steatosis and lipid metabolism in this model. As shown in Fig. 3A, B, tranilast dose-dependently reduced HFHC diet-induced liver weight and liver-to-body weight ratio. No significant changes were observed in NCD-fed mice, indicating that tranilast did not affect basal metabolic processes under normal physiological conditions.

Additionally, tranilast treatment significantly decreased hepatic lipid levels and steatosis compared to the HFHC diet-challenged mice, as shown by reduced hepatic TC and TG levels (Fig. 3C) and lower serum TC, TG, and LDL-C levels (Supplemental Fig. S3A). Histological analysis revealed that tranilast alleviated hepatocyte ballooning and vacuolation, and reduced lipid droplet accumulation, as observed through H&E (Fig. 3D) and ORO staining (Fig. 3E). Furthermore, tranilast effectively altered fatty acid synthesis, uptake, oxidation, chain elongation, and transport in the liver tissues of HFHC diet-fed mice (Fig. 3F and Supplemental Fig. S3B–F). Immunoblot analysis also confirmed that tranilast inhibited the expression of PPARγ, ACLY, and FASN in the liver tissues of HFHC diet-fed mice (Fig. 3G). Collectively, these findings demonstrate that tranilast protects against HFHC diet-induced hepatic steatosis in mice.

### Tranilast ameliorates hepatic inflammation and fibrosis in HFHC diet-fed mice

**Fig. 4:**
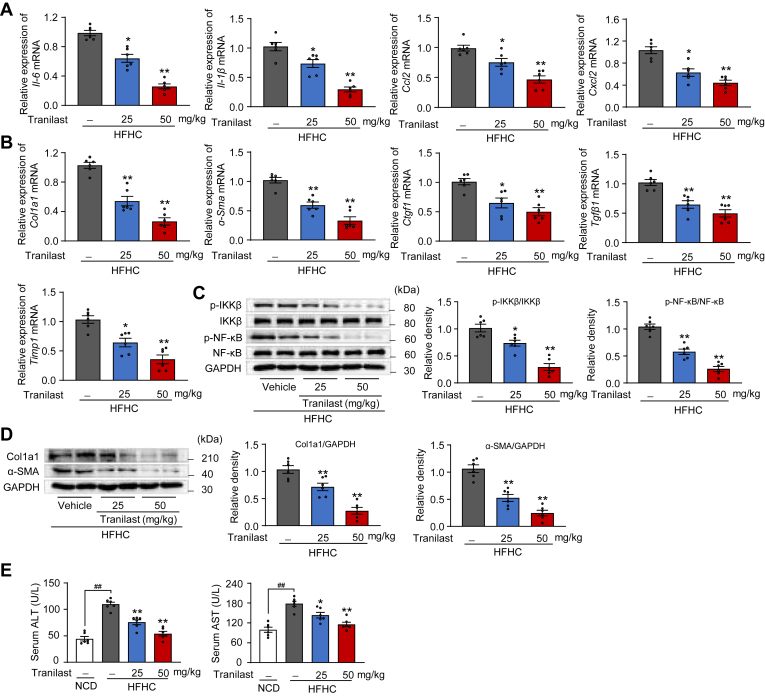
Tranilast ameliorates hepatic inflammation and fibrosis in HFHC diet-fed mice. Same mice as Fig. 2 were used. A, B: Relative mRNA levels of inflammatory genes (A) and profibrotic genes (B) in the liver sections of the indicated mice. C, D: Immunoblot analysis of the indicated proteins for the inflammation signaling (C) and fibrosis pathway (D) in liver tissues of the indicated mice. E: Serum ALT and AST levels of mice in the indicated groups. Data are presented as mean ± SEM (n = 6). ^##^*P* < 0.01, ∗*P* < 0.05, ∗∗*P* < 0.01 *vs*. the indicated groups or the HFHC treatment group. One-way ANOVA was used for statistical analysis (A–E). ALT, alanine aminotransferase; AST, aspartate aminotransferase; HFHC, high-fat high-cholesterol.

Low-grade chronic inflammation is a hallmark of hepatic lipid response and contributes to hepatic wound healing, fibrosis, and exacerbates hepatic lipid accumulation and failure (30). Therefore, we investigated the inhibitory effects of tranilast on NASH-induced liver inflammation and fibrosis in HFHC diet-fed mice. As anticipated, tranilast significantly reduced the mRNA levels of inflammatory mediators (*Il-6*, *Il-1β*, *Ccl2*, and *Cxcl2*) (Fig. 4A) and profibrotic genes (*Col1a1*, *α-Sma*, *Ctgf*, *Tgfβ1*, and *Timp1*) (Fig. 4B) in the liver tissues of HFHC diet-fed mice. Immunoblot analysis and histological staining further confirmed that tranilast markedly reduced inflammatory signaling and pro-fibrotic factors associated with collagen deposition (Fig. 4C, D and Supplemental Fig. S4A, B). We also assessed the role of liver-resident Kupffer cells by examining inflammatory infiltrates in the liver. Remarkably, tranilast normalized the markers of liver-resident Kupffer cells (Supplemental Fig. S4C). Next, we examined whether tranilast could reverse liver injury biomarkers in HFHC diet-fed mice. Tranilast significantly reduced serum ALT and AST activities in these mice (Fig. 4E). Collectively, these findings demonstrate that tranilast protects against HFHC diet-induced NASH progression-associated hepatic inflammation and fibrosis.

### Tranilast attenuates hepatic steatosis, inflammation, and fibrosis in MCD diet-fed mice

**Fig. 5:**
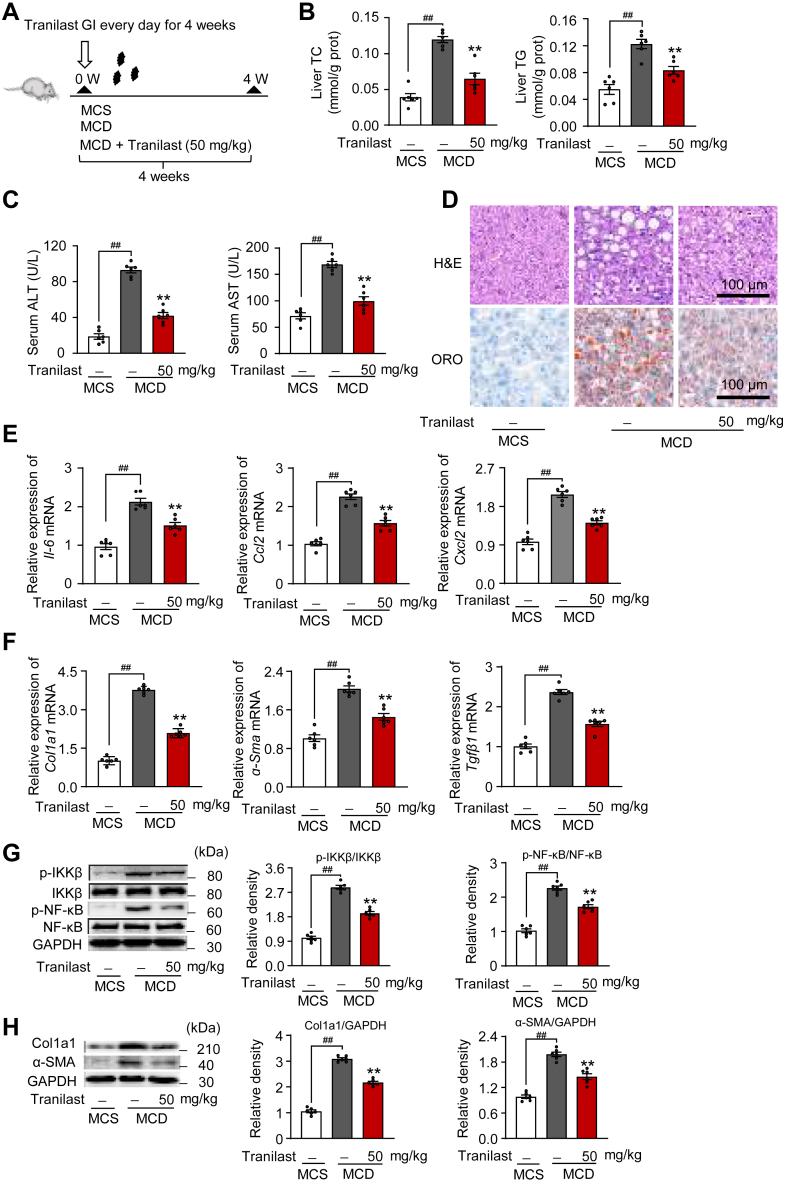
Tranilast attenuates hepatic steatosis, inflammation, and fibrosis in MCD diet-fed mice. 8-week-old male C57BL/6J mice were randomly grouped (n = 6). Mice were given ad libitum access to water and different types of diets. Vehicle or tranilast (50 mg/kg) was administrated to mice by gastric irrigation (GI) once daily for 4 weeks A: Schematic representation of the protocol. B: Levels of TC and TG in the liver tissues. C: Serum ALT and AST levels of the mice in the indicated groups. D: Representative images of H&E and ORO staining in liver sections. Scale bar, 100 μm. E, F: Relative mRNA levels of inflammatory genes (E) and profibrotic genes (F) in liver sections of the indicated mice. G, H: Immunoblot analysis of the indicated proteins for the inflammation signaling (G) and fibrosis pathway (H) in the liver tissues of the indicated mice. Data are presented as mean ± SEM (n = 6). ^##^*P* < 0.01, ∗∗*P* < 0.01 *vs*. the indicated groups or the MCD treatment group. One-way ANOVA was used for statistical analysis (B, D, and E-H). MCD, methionine-choline deficient; MCS, methionine- and choline-supplemented control; ORO, oil red O; TG, triglyceride; TC, total cholesterol.

To further explore the direct hepatic effects of tranilast on lipid metabolism, we established a classical MCD dietary model in mice for 4 weeks (9). The daily oral administration of tranilast for 4 weeks to MCD diet-fed mice is illustrated in the schematic representation (Fig. 5A). Consistent with the results from the HFHC diet-fed model, MCD diet-fed mice showed a significant reduction in serum TC and TG levels, which was reversed by tranilast treatment (Supplemental Fig. S5A). In contrast, tranilast effectively inhibited the increase in TC and TG content in the liver tissues of MCD diet-fed mice (Fig. 5B). As expected, tranilast also reduced serum ALT and AST activities in mice subjected to the MCD diet challenge (Fig. 5C). To assess liver histological changes, we performed hepatic H&E and ORO staining. As shown in Fig. 5D, tranilast significantly reduced macrovesicular steatosis and lipid deposition in the liver tissues of MCD diet-fed mice.

Next, we examined the effects of tranilast on hepatic inflammation and collagen deposition in the liver of MCD diet-fed mice. Immunofluorescence staining and qPCR analysis revealed that tranilast substantially reduced hepatic inflammation, as indicated by the downregulation of Kupffer cells markers (Supplemental Fig. S5B, C), and by the decreased mRNA levels of inflammation-related genes (*Il-6*, *Ccl2*, and *Cxcl2*) (Fig. 5E). Additionally, tranilast effectively alleviated the gene expression of pro-fibrotic genes (*Col1a1*, *α-Sma*, and *Tgfβ1*) in the liver tissues of MCD diet-fed mice (Fig. 5F). Consistent with these results, tranilast also markedly inhibited the MCD diet-induced expression of p-IKKβ, p-NF-κB, α-SMA, and Col1a1 in mice, as shown in Fig. 5G, H. Collectively, these data confirm that tranilast protects against experimental steatohepatitis induced by MCD diet-fed mice.

### Tranilast promotes AMPK activation by inhibiting LKB1 acetylation

**Fig. 6:**
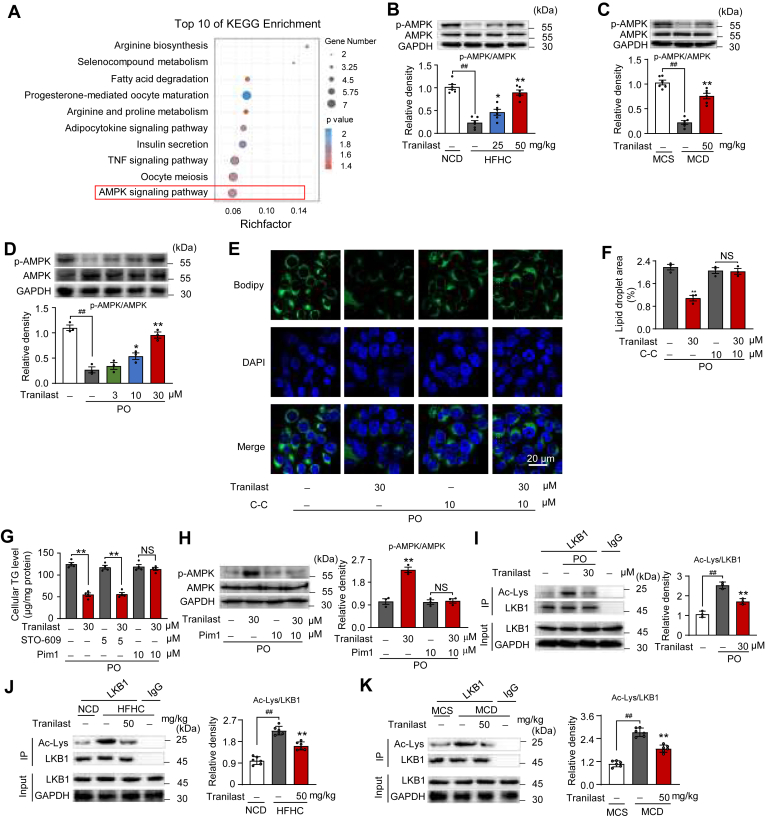
Tranilast promotes AMPK activation by inhibiting LKB1 acetylation. A: Bubble chart showing the KEGG enrichment pathway analysis of DEGs upregulated in liver tissues of the indicated mice. Fisher exact test was used to test the enrichment of DEGs against all identified genes. B: Immunoblot analysis of AMPK phosphorylation from liver tissues of the indicated mice described in Fig. 2. C: Immunoblot analysis of AMPK phosphorylation from the liver tissues of the indicated mice described in Fig. 5. D: Immunoblot analysis of AMPK phosphorylation in primary hepatocytes treated with tranilast in the presence or absence of PO (n = 3). E, F: Representative images of BODIPY staining (E) and quantification of fluorescence (F) in PO-induced L02 cells treated with tranilast in the presence or absence of C-C (10 μM) (n = 3). G: Cellular TG contents in PO-induced L02 cells treated with tranilast in the presence or absence of the CaMKK2 inhibitor (STO-609, 5 μM) or the LKB1 inhibitor (Pim1, 10 μM) (n = 4). H: Immunoblot analysis of AMPK phosphorylation from PO-induced L02 cells treated with tranilast in the presence or absence of Pim1 (n = 3). I: IP and immunoblot analysis of LKB1 acetylation in PO-stimulated L02 cells treated with tranilast (n = 3). J, K: IP and immunoblot analysis of LKB1 acetylation in liver tissues of indicated mice. Data are presented as mean ± SEM (n = 6). ∗∗*P* < 0.01 *vs*. the indicated groups or the HFHC/MCD/PO treatment group. NS, not significant. One-way ANOVA was used for statistical analysis (B-D, I-K). Student's *t* test and Mann-Whitney U test were applied in (F–H). C-C, Compound C; DEGs, differentially expressed genes; HFHC, high-fat high-cholesterol; KEGG, Kyoto encyclopedia of genes and genomes; MCD, methionine-choline deficient; MCS, methionine- and choline-supplemented control; NCD, normal chow diet; PO, palmitic acid and oleic acid; TG, triglyceride.

To investigate the molecular mechanism by which tranilast inhibits hepatic lipid response in NAFLD, we conducted RNA-seq analysis of liver tissues from HFHC-fed mice treated with tranilast. Following the quantification of differentially expressed genes (DEGs), we performed KEGG pathway enrichment analysis. As shown in Fig. 6A, the AMPK signaling pathway emerged as a key pathway involved in various metabolic processes. A heatmap generated from the RNA-seq data revealed that tranilast effectively downregulated the expression of liver genes associated with the AMPK pathway (Supplemental Fig. S6A). Furthermore, preliminary experiments ruled out interference from other pathways.

In support of these findings, immunoblot analysis demonstrated that tranilast significantly promoted AMPK activation both in vivo (Fig. 6B, C) and in vitro (Fig. 6D), as evidenced by increased AMPK phosphorylation in the liver tissues of HFHC/MCD diet-fed mice and PO-induced L02 cells. Immunofluorescence microscopy further confirmed that tranilast substantially reduced PO-induced lipid accumulation, and this effect was reversed by C-C treatment and AMPK inactivation (Fig. 6E, F and Supplemental Fig. S6B), establishing that AMPK activation contributes to the protective effects of tranilast on lipid metabolism. These results prompted us to further investigate how tranilast enhances AMPK activation during hepatic lipid response. As a key sensor of cellular energy status, AMPK is conserved across all eukaryotes (31). AMPK activation is primarily regulated by changes in the AMP/ADP ratio, LKB1, and CaMKK2. As shown in Supplemental Fig. S6C–H, tranilast did not significantly alter the AMP/ATP or ADP/ATP ratios in liver tissues from HFHC and MCD diet-fed mice or in PO-induced hepatocytes. This suggests that tranilast activates AMPK through an AMP/ADP-independent mechanism.

To identify the kinase involved in these processes, we examined the effects of tranilast on PO-induced cellular TG accumulation in the presence or absence of STO-609 (a CaMKK2 inhibitor) or Pim1 (a LKB1 inhibitor) were examined. Surprisingly, only Pim1 treatment diminished the inhibitory effects of tranilast on cellular TG accumulation in PO-induced L02 cells (Fig. 6G), suggesting that LKB1 plays a role in lipid metabolism. Consistent with this, tranilast’s promotion of AMPK phosphorylation was largely counteracted by Pim1 treatment (Fig. 6H), confirming the involvement of the LKB1-AMPK axis in tranilast’s beneficial effects on lipid accumulation. We then explored the potential mechanism by which tranilast modulates LKB1 activity. Recent evidence suggests that LKB1 acetylation is critical for AMPK activation under pathological conditions (32). Immunoprecipitation data revealed that tranilast significantly reduced PO-induced LKB1 acetylation (Fig. 6I), and similar reductions were observed in liver tissues from HFHC or MCD diet-fed mice (Fig. 6J, K), indicating that tranilast deacetylates LKB1 and thereby activates AMPK. Furthermore, the inhibitory effects of tranilast on PO-induced TG levels and AMPK activation were reversed by LKB1 knockdown and constitutive acetylation of LKB1 through mutation (Supplemental Fig. S6I, J). In summary, these findings demonstrate that tranilast activates AMPK through the inhibition of LKB1 acetylation.

### AMPK is essential for the inhibitory effects of tranilast on the hepatic lipid response

**Fig. 7:**
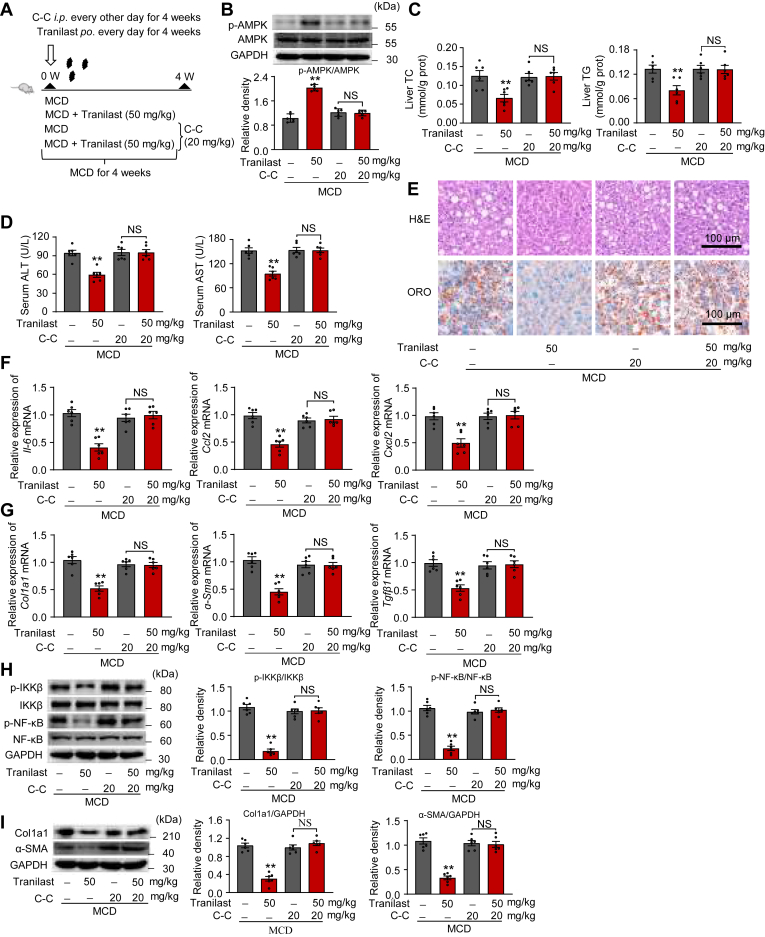
AMPK is essential for the inhibitory effects of tranilast on the hepatic lipid response. 8-week-old male C57BL/6J mice were ad libitum grouped (n = 6 per group) and fed an MCD diet for 4 weeks. Mice were administrated to vehicle or tranilast (50 mg/kg) by gastric irrigation (GI) once daily for 4 weeks and were injected *i.p.* with either vehicle or C-C (20 mg/kg) every 2 days for 4 weeks. A: Schematic representation of the protocol. B: Immunoblot analysis confirmed the targeting of AMPK phosphorylation in liver sections of the indicated mice. C: Levels of TC and TG in liver tissues. D: Serum ALT and AST levels of mice in the indicated groups. E: Representative images of H&E and ORO staining in liver sections. Scale bar, 100 μm. F, G: Relative mRNA levels of inflammatory genes (F) and profibrotic genes (G) in liver sections of the indicated mice. H, I: Immunoblot analysis of the indicated proteins for the inflammation signaling (H) and fibrosis pathway (I) in liver tissues of the indicated mice. Data are shown as mean ± SEM (n = 6). ∗∗*P* < 0.01 *vs*. the indicated groups or the MCD treatment group. NS, not significant. Student's *t* test and Mann-Whitney U test were used for statistical analysis (B-D, F-I). ALT, alanine aminotransferase; AST, aspartate aminotransferase; C-C, Compound C; MCD, methionine-choline deficient; ORO, oil red O.

To determine whether the AMPK pathway is essential for tranilast’s inhibitory effects on hepatic lipid response in vivo, we established a well-characterized hepatic lipid response model. Mice were fed an MCD diet and administered either vehicle or tranilast via oral gavage, alongside an intraperitoneal injection of C-C (Fig. 7A). As shown in Fig. 7B, C-C treatment for 2 weeks effectively blocked AMPK activation in liver tissues. As expected, C-C treatment largely reversed the beneficial effects of tranilast on MCD diet-induced serum and liver lipid levels, as well as liver damage. This was evidenced by increases in serum TC and TG levels (Supplemental Fig. S7), liver TC and TG levels (Fig. 7C), plasma ALT and AST activities (Fig. 7D), and hepatic H&E pathological and ORO staining (Fig. 7E). Furthermore, C-C treatment almost completely abolished the beneficial effects of tranilast on hepatic inflammation and collagen deposition in MCD diet-fed mice. These changes were reflected in qPCR assays (Fig. 7F, G) and immunoblot analysis (Fig. 7H, I). Collectively, these results suggest that tranilast’s inhibitory effects on hepatic lipid response in NAFLD are primarily mediated through AMPK activation.

## Discussion

**Fig. 8:**
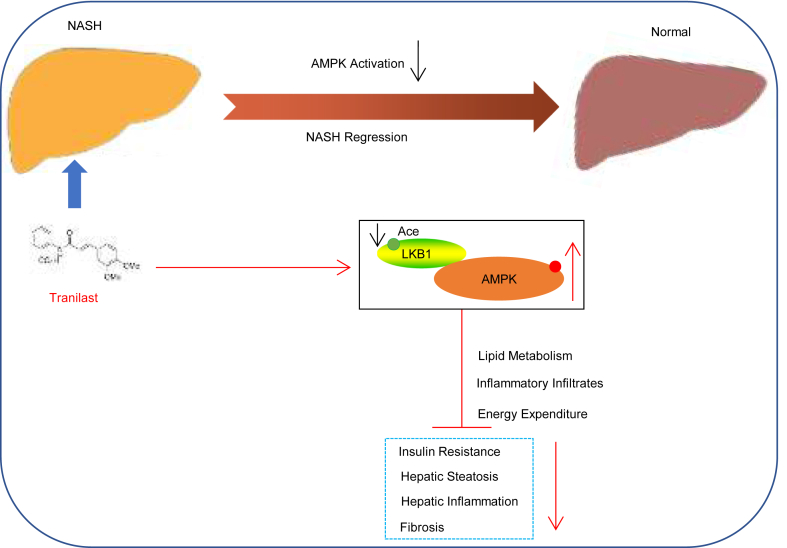
Proposed schematic illustration of a potential mechanism of tranilast in combatting the hepatic lipid response in NAFLD. Tranilast alleviates the hepatic lipid response in NAFLD by deacetylating LKB1 and promoting AMPK activation, which contributes to preventing hepatic steatosis, inflammation, and fibrosis. AMPK, adenosine monophosphate (AMP)-activated protein kinase; LKB1, liver kinase B1; NAFLD, nonalcoholic fatty liver disease.

Given the increasing prevalence of NASH and the lack of approved treatments despite numerous attempts, identifying new drug candidates for intervention is a critical clinical need. In this study, we identified the anti-allergic drug tranilast as a potential therapeutic agent for modulating hepatic lipid metabolism through compound screening. A previous study demonstrated that tranilast improves hepatic fibrosis in a rat model of NASH by downregulating TGF-β in liver-resident Kupffer cells (26). However, the effects of tranilast on obesity and its ability to prevent the progression from NAFL to NASH, as well as its role in hepatocytes, have not been reported. Moreover, the molecular mechanisms underlying tranilast’s effects remain unclear. In the present work, we examined the therapeutic effects of tranilast on hepatic lipid metabolism in NAFLD, specifically in models of HFHC and MCD diet-induced liver injury and clarified its underlying molecular mechanisms. The proposed molecular mechanism by which tranilast modulates hepatic lipid metabolism is shown in Fig. 8. Our findings suggest that tranilast represents a promising novel therapeutic strategy for targeting LKB1 deacetylation in NAFLD.

Here, we identified tranilast as a repurposed drug candidate targeting hepatic lipid response using our optimized in-house library. Tranilast effectively attenuated lipid accumulation and inflammatory responses in vitro. We then demonstrated that tranilast reduced liver inflammation, steatosis, and fibrosis in nutritional models of hepatic lipid response induced by HFHC and MCD diets. While this phenomenon has received limited investigation, its underlying mechanisms and physiological relevance remain unclear. To address this, we explored the molecular mechanism of tranilast in modulating hepatic lipid response in NAFLD. Our findings suggest that tranilast reduces liver steatosis and inflammation, presenting a compelling strategy to mitigate hepatic lipid response. These results further position tranilast as a promising therapeutic candidate for managing hepatic lipid dysregulation in NAFLD. Regarding the PRESTO trial, which reported evidence of liver damage following systemic administration of tranilast (33), the observed toxicity was likely due to the administration of very high dosages. In our study, we used 25 or 50 mg/kg of tranilast daily, corresponding to a human equivalent dose of approximately 2.1 or 4.1 mg/kg/day. Compared to the significantly higher dosages reported in the trial, our results highlight the safety of tranilast at these lower, clinically relevant doses.

With the exception of liver-resident Kupffer cells, hepatocytes are the most well-characterized liver cells, crucial for the liver's physiological and metabolic functions. Our study highlighted that tranilast specifically targets hepatocytes, which store TGs and lipids in NAFLD. Hepatic lipid droplets and steatosis are exacerbated by the increased uptake and synthesis of excess lipids (34). Inflammation plays a central role in both the initiation and the progression of hepatic lipid response in NAFLD, driving the release of proinflammatory cytokines and causing hepatocyte damage. In this study, tranilast reduced hepatic lipid content in PO-induced L02 cells and primary hepatocytes. Specifically, tranilast inhibited the mRNA expression of *IL-6*, *TNF-*α, and *IL-1β* in vitro. These findings provide strong evidence that tranilast suppresses hepatic lipid accumulation and inflammatory responses in vitro.

HFHC diets consistently induce NAFLD in mice, as shown by the development of obesity, hyperlipidemia, insulin resistance, significant inflammation, and partial fibrosis (35). In this study, we employed a series of well-established HFHC diet-fed mouse models, demonstrating that tranilast reduced body weight, fasting serum glucose and insulin concentrations, and various biochemical parameters. Moreover, tranilast significantly decreased HFHC diet-induced liver weight, the liver-to-body weight ratio, liver lipid accumulation, and serum triglyceride (TG), total cholesterol (TC), and low-density lipoprotein cholesterol (LDL-C) concentrations. Our results also demonstrated that tranilast administration alleviated hepatic steatosis in mice induced by the HFHC diet. In addition to steatosis, liver inflammation and fibrosis persist throughout the progression of the hepatic lipid response in NAFLD. Among the various inflammatory pathways and hepatic fibrogenic markers identified, the NF-κB pathway and fibrogenic proteins are the most extensively discussed in the context of HFHC diet feeding (6, 7). Furthermore, accumulating evidence suggests that the inhibition of NF-κB and the antifibrotic effects associated with it play a crucial role in mitigating the hepatic lipid response (35). In our study, tranilast inhibited the HFHC diet-induced IKKβ/NF-κB pathway and collagen deposition in mice, further supporting the significant ameliorative effects of tranilast on the hepatic lipid response. Based on these findings, targeting hepatic inflammation and fibrosis with tranilast may represent a potential therapeutic strategy for preventing and treating diet-induced metabolic liver diseases.

The MCD diet model induces hepatic steatosis similar to the pathological characteristics of NASH in humans (36). In the NASH model induced by the MCD diet, impaired VLDL secretion blocks TG transport from the liver to plasma, leading to a marked increase in hepatic TG levels and a reduction in plasma TG levels. In this study, we extended our investigations to the MCD model and demonstrated that tranilast treatment inhibited hepatic TG production while increasing plasma TG levels in MCD diet-fed mice. Furthermore, tranilast significantly improved MCD diet-induced hepatic steatosis and inflammation. Additionally, tranilast substantially reduced hepatic fibrosis in this model, providing compelling evidence that tranilast can effectively mitigate the hepatic lipid response. Our findings highlight that tranilast protects against the hepatic lipid response in NAFLD induced by the MCD diet.

To elucidate the mechanism by which tranilast inhibits hepatic steatosis, inflammation, and fibrosis induced by HFHC and MCD diets, KEGG pathway enrichment analysis of RNA-seq data revealed that tranilast exerts its effects primarily through the activation of the AMPK pathway. Notably, tranilast does not alter metabolic parameters during lean chow feeding, highlighting its specific effects on the hepatic lipid response. This finding aligns with previous studies conducted in rat models (26). In recent years, AMPK has emerged as a well-established molecular target for treating metabolic disorder-related diseases (37, 38). AMPK acts as a nutrient and energy sensor, regulating energy homeostasis in response to diverse stimuli (39). Structurally, AMPK is a heterotrimer consisting of a catalytic α-subunit and regulatory β- and γ-subunits, and its activity is inversely correlated with hepatic lipid accumulation (40).

Numerous studies have shown that hepatic AMPK loss-of-function exacerbates diet-induced hepatic lipid pathology, while its gain-of-function mitigates these effects (41, 42). Given the critical role of AMPK in regulating NAFLD, our findings suggest that targeting the deacetylation of LKB1 by tranilast represents a promising therapeutic strategy for metabolic liver diseases. Previous research indicates that LKB1 signaling plays an essential role in the progression of NASH and hepatocyte death (43), further underscoring the link between LKB1 and NAFLD. Our study confirms that deacetylation of LKB1 is pivotal to the inhibitory effects of tranilast on the hepatic lipid response. These results align with prior evidence that liver-specific activation of AMPK can effectively suppress hepatic lipid response in NAFLD (42). In this study, we further clarified the mechanism by demonstrating that tranilast promotes AMPK activation by inhibiting LKB1 acetylation.

Our study has several limitations. First, it primarily focuses on the liver, and additional investigations in other tissues are warranted. Second, mechanistic studies are needed to clarify the precise mechanism by which tranilast inhibits LKB1 acetylation. Finally, these findings should be validated in rat and macaque models.

## Conclusion

In conclusion, our study demonstrates that tranilast, an approved anti-allergic drug, prevents the hepatic lipid response in NAFLD through LKB1 deacetylation. These findings provide strong experimental evidence supporting the therapeutic potential of deacetylating LKB1 for treating NASH. More importantly, tranilast may represent a novel agent with significant translational potential for the pharmacological management of metabolic diseases.

## Data availability

All data are contained within the manuscript and supplementary data, except that RNA-seq data have been deposited into the Gene Expression Omnibus database (accession no. GSE249350).

## Supplemental data

This article contains supplemental data.

## Conflict of interests

The authors declare that they have no conflicts of interest with the contents of this article.
