# Supplementary material for: Small molecule-driven LKB1 deacetylation is responsible for the inhibition of hepatic lipid response in NAFLD
Source: J Lipid Res. 2025 Jan 2;66(2):100740. doi: 10.1016/j.jlr.2024.100740 (PMC11808498; doi:10.1016/j.jlr.2024.100740)
Supplement: Supplementary Tables and Figures [file mmc1.doc]

**Table S1. Sequences of the primers used for real-time PCR.**

| **Gene** | **Forward (5’ to 3’)** | **Reverse (5’ to 3’)** |
| --- | --- | --- |
| *Fasn* mouse | CTGCGGAAACTTCAGGAAATG | GGTTCGGAATGCTATCCAGG |
| *Cd36* mouse | GACTGGGACCATTGGTGATGA | AAGGCCATCTCTACCATGCC |
| *Pparg* mouse | ATTCTGGCCCACCAACTTCGG | TGGAAGCCTGATGCTTTATCCCCA |
| *Srebf1c* mouse | CACTTCTGGAGACATCGCAAAC | ATGGTAGACAACAGCCGCATC |
| *Fabp1* mouse | TGGTCCGCAATGAGTTCACCCT | CCAGCTTGACGACTGCCTTGACTT |
| *Acaca* mouse | GGCCAGTGCTATGCTGAGAT | AGGGTCAAGTGCTGCTCCA |
| *Scd1* mouse | TCTTCCTTATCATTGCCAACACCA | GCGTTGAGCACCAGAGTGTATCG |
| *Il-6* mouse | TAGTCCTTCCTACCCCAATTTCC | TTGGTCCTTAGCCACTCCTTC |
| *Tnf-α* mouse | CATCTTCTCAAAATTCGAGTGACAA | TGGGAGTAGACAAGGTACAACCC |
| *Il-1β* mouse | CCGTGGACCTTCCAGGATGA | GGGAACGTCACACACCAGCA |
| *Ccl2* mouse | TACAAGAGGATCACCAGCAGC | ACCTTAGGGCAGATGCAGTT |
| *Cxcl2* mouse | GCGCCCAGACAGAAGTCATA | CAGTTAGCCTTGCCTTTGTTCA |
| *Timp1* mouse | GAGACCACCTTATACCAGCGTT | TACGCCAGGGAACCAAGAAG |
| *Ctgf* mouse | TGACCCCTGCGACCCACA | TACACCGACCCACCGAAGACACAG |
| *Col1a1* mouse | TGCTAACGTGGTTCGTGACCGT | ACATCTTGAGGTCGCGGCATGT |
| *α-Sma*  mouse | CCCAGACATCAGGGAGTAATGG | TCTATCGGATACTTCAGCGTCA |
| *Tgfβ1* mouse | ATTTGGAGCCTGGACACACA | GAGCGCACAATCATGTTGGA |
| *F4/80* mouse | TTCCTGCTGTGTCGTGCTGTTC | GCCGTCTGGTTGTCAGTCTTGTC |
| *Tlr4* mouse | GAGCCGGAAGGTTATTGTGGTAGTG | AGGACAATGAAGATGATGCCAGAGC |
| *Tlr9* mouse | GAGCCTGAGCCACACCAACATC | CTTGTAGTAGCAGTTCCCGTCCATG |
| *Pparα*  mouse | AGAGCCCCATCTGTCCTCTC | ACTGGTAGTCTGCAAAACCAAA |
| *Cpt1α* mouse | AGATCAATCGGACCCTAGACAC | CAGCGAGTAGCGCATAGTCA |
| *Hacd2* mouse | TCTTTCCAGGTGATGTCAAGAG | CCAGGCAATGACAAACAGAAG |
| *Elovl6* mouse | CATGCCGTCATGTACTCTTACT | CATCTGAGTGATCTGGGACAAG |
| *Slc27a5* mouse | CACCCCCAGGGCTACGCT | CAGTGCTTGCCGCTCTAAA |
| *Pgc-1α* mouse | ATACCGCAAAGAGCACGAGAAG | CTCAAGAGCAGCGAAAGCGTCACAG |
| *G6Pase* mouse | CGACTCGCTATCTCCAAGTGA | GTTGAACCAGTCTCCGACCA |
| *Pepck* mouse | AAGCATTCAACGCCAGGTTC | GGGCGAGTCTGTCAGTTCAAT |
| *β-Actin* mouse | GTGACGTTGACATCCGTAAAGA | GCCGGACTCATCGTACTCC |
| *IL-6* human | GAGTAGTGAGGAACAAGCCAGA | AAGCTGCGCAGAATGAGATGA |
| *TNF-ɑ* human | TGGCGTGGAGCTGAGAGATA | TGATGGCAGAGAGGAGGTTG |
| *IL-1β* human | GCCAGTGAAATGATGGCTTATT | AGGAGCACTTCATCTGTTTAGG |
| *β-Actin human* | CATGTACGTTGCTATCCAGGC | CTCCTTAATGTCACGCACGAT |


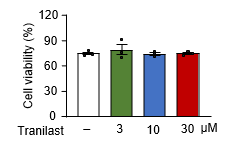


**Supplemental Figure S1.** Cell viability detected by CCK8 analysis in the indicated groups (n = 3). One-way ANOVA was used for statistical analysis.


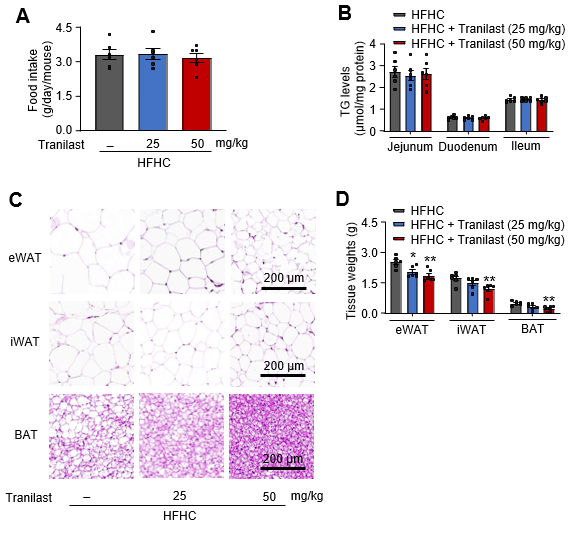


**Supplemental Figure S2.** Effects of tranilast on food intake, absorption, and adipose tissue described in **Fig.2**. Food intake (**A**) and absorption (**B**) of the indicated mice. H&E analysis (**C**) and weights (**D**) of various adipose tissues (eWAT, iWAT, and BAT) of the indicated mice. Scale bar, 200 µm. Data are presented as mean ± SEM (n = 6). **p* < 0.05, ***p* < 0.01 *vs*. the HFHC treatment group. One-way ANOVA was used for statistical analysis (A, B, and D).


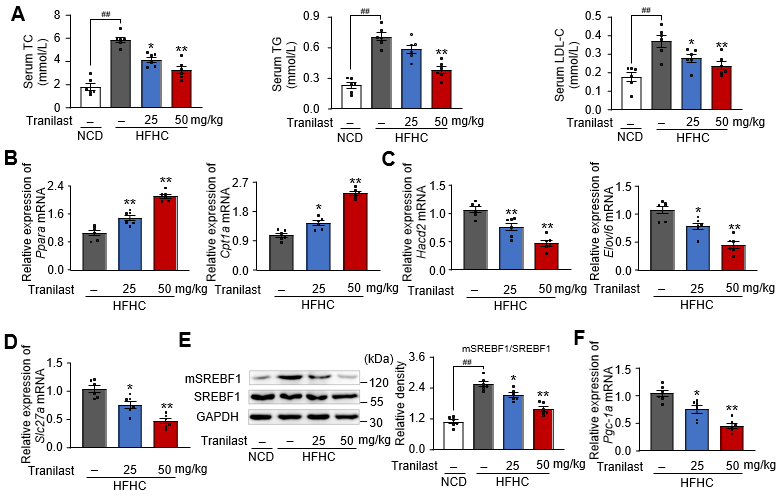


**Supplemental Figure S3.** Effects of tranilast on serum lipid contents and fatty acid metabolism-related genes and SREBF1 from liver tissues described in **Fig.2.** (**A**) Levels of TC, TG, and LDL-C in serum were determined in the indicated mice. (**B**-**F**) Fatty acid metabolism-related genes and SREBF1 were measured in the indicated mice. Data are presented as mean ± SEM (n = 6). ^##^*p* < 0.01, **p* < 0.05, ***p* < 0.01 *vs*. the indicated groups or the HFHC treatment group. One-way ANOVA was used for statistical analysis (A-F).


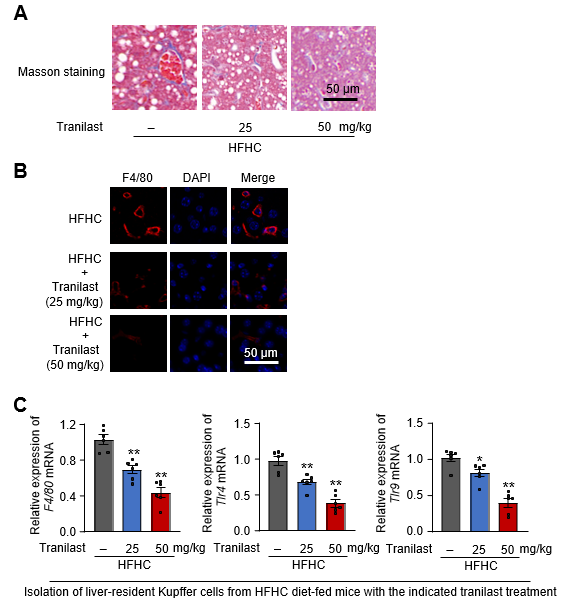


**Supplemental Figure S4.** Tranilast limits hepatic inflammation in the liver sections of the indicated mice described in **Fig.2.** Masson's trichrome staining (**A**) and immunofluorescence staining of F4/80-positive cells (**B**) in liver tissues of indicated mice. Scale bar, 50 µm. (**C**) Hepatic gene expression of Kupffer cells. Data are presented as mean ± SEM (n = 6). **p* < 0.05, ***p* < 0.01 *vs*. the HFHC treatment group. One-way ANOVA was used for statistical analysis (C).


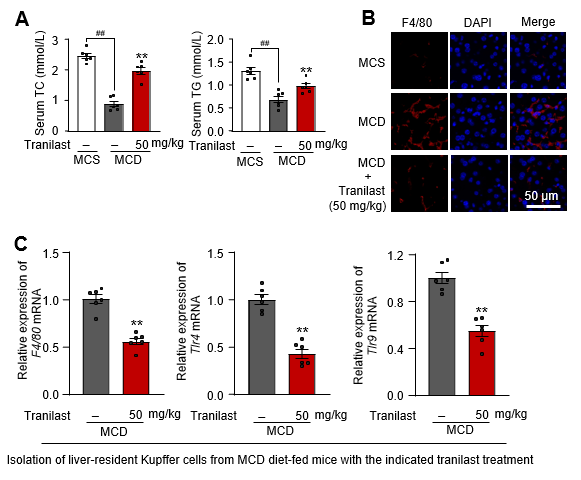


**Supplemental Figure S5.** Effects of tranilast on serum lipid contents and hepatic inflammation in liver sections of the indicated mice described in **Fig.5.** (**A**) Levels of TC and TG in serum. (**B**) Immunofluorescence staining of F4/80-positive cells in liver tissues of indicated mice. Scale bar, 50 µm. (**C**) Hepatic gene expression of Kupffer cells. Data are presented as mean ± SEM (n = 6). ^##^*p* < 0.01, ***p* < 0.01 *vs*. the indicated groups or the MCD treatment group. One-way ANOVA was used in (A). Student's *t*-test and Mann-Whitney U test were applied in (C).


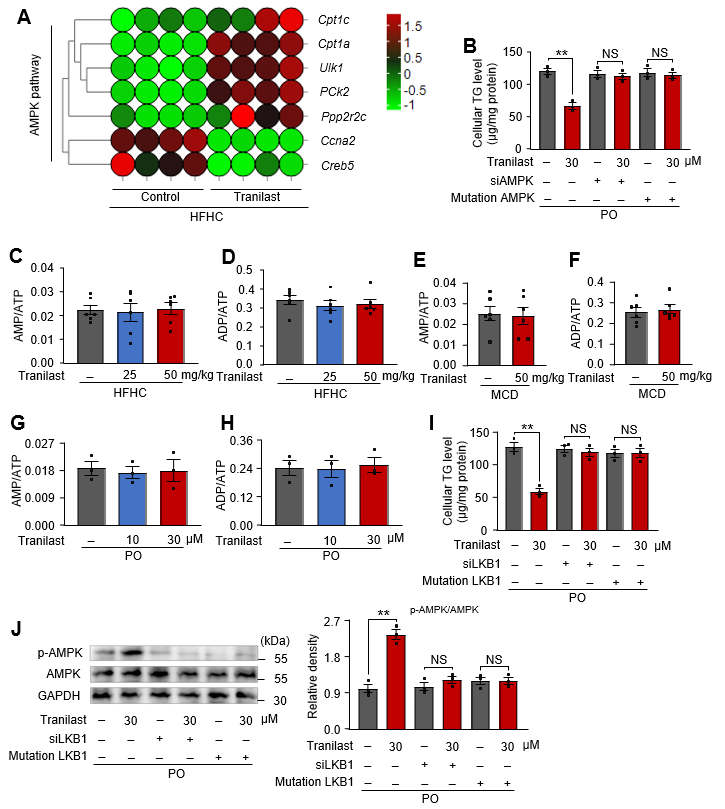


**Supplemental Figure S6.** (**A**) Heatmap of AMPK pathway-related hepatic gene expression profiles based on the RNA-Seq dataset. (**B**) Effects of tranilast on TG content in inactivation AMPK by knockdown AMPK with siRNA and mutation of AMPK. (**C**-**H**) Effects of tranilast on AMP/ATP and ADP/ATP in liver tissues of indicated mice described in **Fig.2** and **Fig.5**, and PO-induced hepatocytes. (**I**, **J**) Effects of tranilast on PO-induced TG levels (**I**) and AMPK inactivation (**J**) by LKB1 knockdown and acetylation of LKB1 with mutation. Data are presented as mean ± SEM (n = 6). ***p* < 0.01 *vs*. the indicated groups. NS, not significant. One-way ANOVA was used in (C, D, G, and H). Student's *t*-test and Mann-Whitney U test were applied in (B, E, F, I, and J).


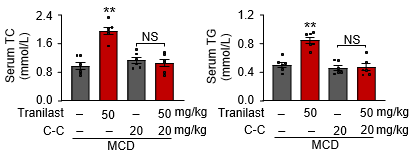


**Supplemental Figure S7.** Effects of tranilast on serum lipid contents described in **Fig.7.** Levels of TC and TG in serum in the indicated mice. Data are presented as mean ± SEM (n = 6). ***p* < 0.01 *vs*. the MCD treatment group. NS, not significant. Student's *t*-test and Mann-Whitney U test were applied for statistical analysis.
